# An Engineered Endomorphin-2 Gene for Morphine Withdrawal Syndrome

**DOI:** 10.1371/journal.pone.0149877

**Published:** 2016-03-22

**Authors:** Fei-xiang Wu, Yan He, Hui-ting Di, Yu-ming Sun, Rui-rui Pan, Wei-feng Yu, Renyu Liu

**Affiliations:** 1 Department of Anesthesiology & Intensive Care, Eastern Hepatobiliary Surgery Hospital, Second Military Medical University, Shanghai, 200438, China; 2 Department of Anesthesiology and Critical Care, Perelman School of Medicine at the University of Pennsylvania, Philadelphia, PA, 19104, United States of America; 3 Department of Anesthesiology, Dongfang Hospital, Fujian, 354200, China; Radboud University Medical Centre, NETHERLANDS

## Abstract

An optimal therapeutics to manage opioid withdrawal syndrome is desired for opioid addiction treatment. Down-regulation of endogenous endomorphin-2 (EM2) level in the central nervous system after continuous morphine exposure was observed, which suggested that increase of EM2 could be an alternative novel method for opioid dependence. As a short peptide, the short half-life of EM2 limits its clinical usage through conventional administration. In the present study, we engineered an EM2 gene using a signal peptide of mouse growth factor for an out-secretory expression of EM2 and an adenovirus as a vector, which ultimately sustained the release of EM-2. After administration of the adenovirus in central nervous system, a sustained increase of EM2 level in the cerebral spinal fluid (CSF) was observed along with a reduction of morphine withdrawal syndrome. These findings suggest that the engineered EM2 gene delivered to the central nervous system could be a novel therapeutics for withdrawal syndrome in opioid dependent subjects.

## Introduction

Opioid dependence affects more than 1 million people in North America alone each year [1, 2]. Opioid dependence associated with various infectious diseases, serious medical complications, overdose mortality, tolerance, loss of social functioning, financial instability, and serious crime [3–6]. Sudden discontinuation or decreased opioid intake leads to withdrawal symptoms including nausea, diarrhea, severe itching, tachycardia, and depression [7, 8]. Current managements of opioid dependence are using full mu opioid receptor (MOR) agonist, methadone, or partial MOR agonist, buprenorphine [9–11]. However, both methadone and buprenorphine are addictive and are ineffective in 15% to 25% patients [12], and a long-term monitored treatment is required due to potential relapse [13].

Endomorphin-2 (EM2) is an endogenous agonist for MOR with high affinity and high selectivity, which has an exciting and promising therapeutic potential for pain or opioid dependence with less deleterious side effects of opiates [14–16]. Spinal EM2 was decreased by the activation of MOR but enhanced by the blockade of spinal MOR, which suggested that EM2 is involved in the pathophysiologic state of dependence after chronic exposure to opioids and could be used as an alternative drug for opioid dependence [17]. However, the usefulness of synthetic EM2 was limited due to its short half-life [18]. The alternative approach is to increase the half-life of EM2 by structural modifications of the EM2 protein [19–21] or using a genetic engineering approach to sustain the release of EM2 [22]. In the present study, we hypothesized that the sustained release of endogenous EM2 by genetic engineering could be used to manage opioid withdrawal syndrome.

## Materials and Methods

### Materials

**Fig 1:**
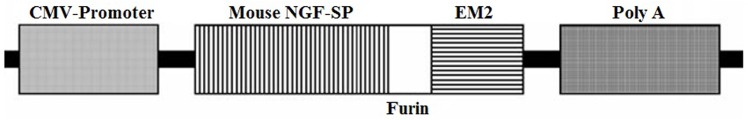
The fusion gene complex for endogenous endomorphin-2 (EM2) consisted of a gene for human EM2 and a gene for a signal peptide of mouse nerve growth factor (NGF). The fusion gene expression cassette was engineered under the control of a cytomegalovirus (CMV) promoter. The white area represented the cleavage site for Furin.

The recombinant adenovirus (Deletion of the E1/E3 regions of genome), Ad-EM2 (an adenovirus with EM2) and Ad-EGFP (an adenovirus with enhanced green fluorescence protein) were produced, purified, and stored as previously described [22, 23]. The titers of the virus were adjusted to 1×10^11^ PFU/ml (PFU = plaque-forming units). The target gene of Ad-EM2 was a fusion gene complex consisting of a gene for human EM2 and a gene for a signal peptide of mouse nerve growth factor (Fig 1). The fusion gene expression cassette was engineered under the control of a cytomegalovirus (CMV) promoter. The signal peptide of mouse growth factor was introduced as an out-secretory peptide for EM2.

### Animals

All animal experiments were carried out under the guidelines issued by the Administrative Committee of Experimental Animal Care and Use of Shanghai, and conformed to the National Institute of Health guidelines on the ethical use of animals. The institutional review committee of Eastern Hepatobiliary Surgery Hospital approved the experimental protocol. Male Sprague-Dawley rats (Weight: 200-250g, Age: 10–12 weeks from Shanghai Experimental Animal Center of Chinese Academy of Sciences) were housed in a pathogen free condition with a 12:12 hour light /dark cycle. The room and cage conditions were monitored twice a day. Monitoring for health problems were performed three times a day. All rats were healthy and no death was found during the experiments. The rats were euthanized by CO_2_ after the whole experiments were finished.

### Animal model and protocol

Rats were divided into four groups randomly (n = 10/group): an Ad-EM2 group (Ad-EM2 with titers of 5×10^9^ PFU); an Ad-EGFP group (Ad-EGFP with titers of 5×10^9^ PFU); a NS group (Normal Saline injection); and a sham group. The morphine dependent model was constructed by subcutaneous administration of daily escalating doses of morphine three times/day for 6 consecutive days (5, 10, 20, 40, 50, 60 mg/kg) [24]. The same volumes of normal saline (NS) were received subcutaneously in the sham group. In the Ad-EM2 group and Ad-EGFP group, dependent rats received intrathecal 50μl of the various virus solutions after the last dosage of morphine was administered. In the NS group and sham group, the same volumes of NS were injected intrathecally.

### Lumbar subarachnoid catheterization

Lumbar subarachnoid catheterization was carried out 3 days before starting the morphine administration. Rats were anesthetized with intraperitoneal injection of 40 mg/kg of sodium pentobarbital (Chemical Reagent Company, Shanghai, China). The vital signs of rats were monitored by the MouseOx^®^ Plus pulse oximeter (Starr Life Sciences Corporation, USA). A sterile PE10 polyethylene catheter (Becton Dickinson, Sparks, MD, USA) was used for intrathecal placement between lumbar vertebrae 5(L5) and lumbar vertebrae 6 (L5) [25]. The external part of the indwelling catheter was protected under the skin to the cervical region according to the Milligan’s method [26]. A lidocaine test was performed to determine the position of the catheter. The catheter was utilized to deliver the virus and to collect cerebrospinal fluid (CSF) samples. On the 6th day of establishment of the morphine dependent model, the Ad-EM2, Ad-EGFP or NS were delivered through the catheters after the last dosage of morphine. Before injection of the virus and on each of the 1st, 3rd, 5th, 7th, and 10th day after injection of the virus, the CSF samples were collected.

### The scores of the withdrawal syndrome

On the 1st, 3rd, 5th, 7th, and 10th day after intrathecal delivery of the adenovirus carried gene, morphine withdrawal syndrome was induced by intraperitoneal injection of naloxone (2mg/kg). The symptoms of morphine withdrawal syndrome in each animal were observed for 30 min after naloxone injection. The scores of withdrawal symptoms were determined according to Maldonado's modified method as described in our previous study (Table 1) [27, 28]. The body weight was measured after scoring.

**Table 1:** Scores of the symptoms of morphine withdrawal syndrome.

Symptoms	Scores		
	1	2	3
wet dog shake	1–3 times	4-6times	≥7times
writhing	1-3times	4-6times	≥7times
teeth chattering	1-3times	4-6times	≥7times
jumping	1-3times	4-6times	≥7times
rearing	1-3times	4-6times	≥7times
body grooming	1-3times	4-6times	≥7times
ptosis	1-4times	5-8times	≥9times

### ELISA assay

The EM2 concentration in CSF was measured using a sandwich ELISA kit (Uscn Life Science Inc, Wuhan, China) according to the manufacturer’s protocol. 96-well micro-titers were used for incubation and measurement. The optical density of each sample was read at 450 nm by a microplate reader (Bio-Rad Laboratories, Shanghai, China). Endomorphin-2 concentration was determined according to the standard curve as described previously [22].

### Green fluorescence assay

On the 10th day after intrathecal injection, rats were anesthetized with intraperitoneal injection of 40 mg/kg of sodium pentobarbital and perfused through the ascending aorta with saline, followed by 4% paraformaldehyde in 0.16 M phosphate buffer (pH 7.2–7.4) containing 1.5% picric acid. After perfusion, the tissues of L5 spinal cord were collected and fixed in the same fixation solution for 3 h and then in 15% sucrose overnight. Transverse spinal sections (30μm) were obtained on a cryostat and processed for EGFP expression estimation with a green fluorescence assay. These sections were examined under an Olympus fluorescence microscope (Olympus, Japan).

### Statistical analysis

All data were presented as mean ± SEM. Statistical analysis was carried out using two-way ANOVA followed by post-hoc Tukey's multiple comparison using GraphPad Prism software (Version 5. GraphPad Software Inc, CA, USA). P<0.05 was considered statistically significant.

## Results

### The persistent increase of EM2 in CSF

**Fig 2:**
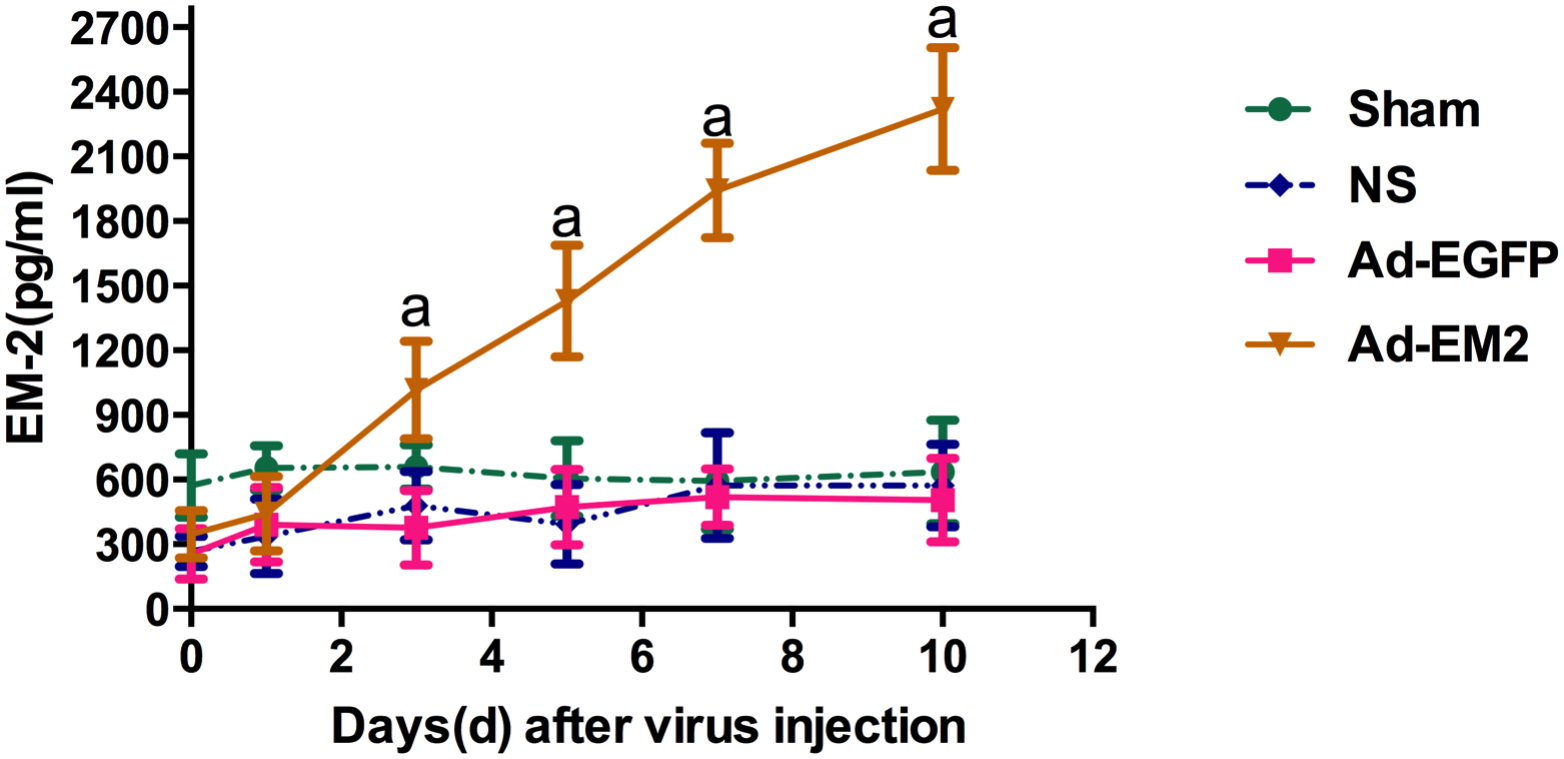
Endomorphin-2 (EM2) expression in CSF after injection. On the 3rd, 5th, 7th, and 10th day after intrathecal injection, the EM2 in cerebral spinal fluid (CSF) increased significantly in the Ad-EM2 groups compared to that in the Ad-EGFP group and NS group (^a^P<0.0001). Endomorphin-2 was indicated as EM2; adenovirus with EM2 was indicated as Ad-EM2; adenovirus with enhanced green fluorescence protein was indicated as Ad-EGFP; normal saline group with subcutaneous morphine injection and intrathecal NS administration was indicated as NS; Sham group with subcutaneous NS injection and intrathecal NS administration was indicated as Sham.

**Fig 3:**
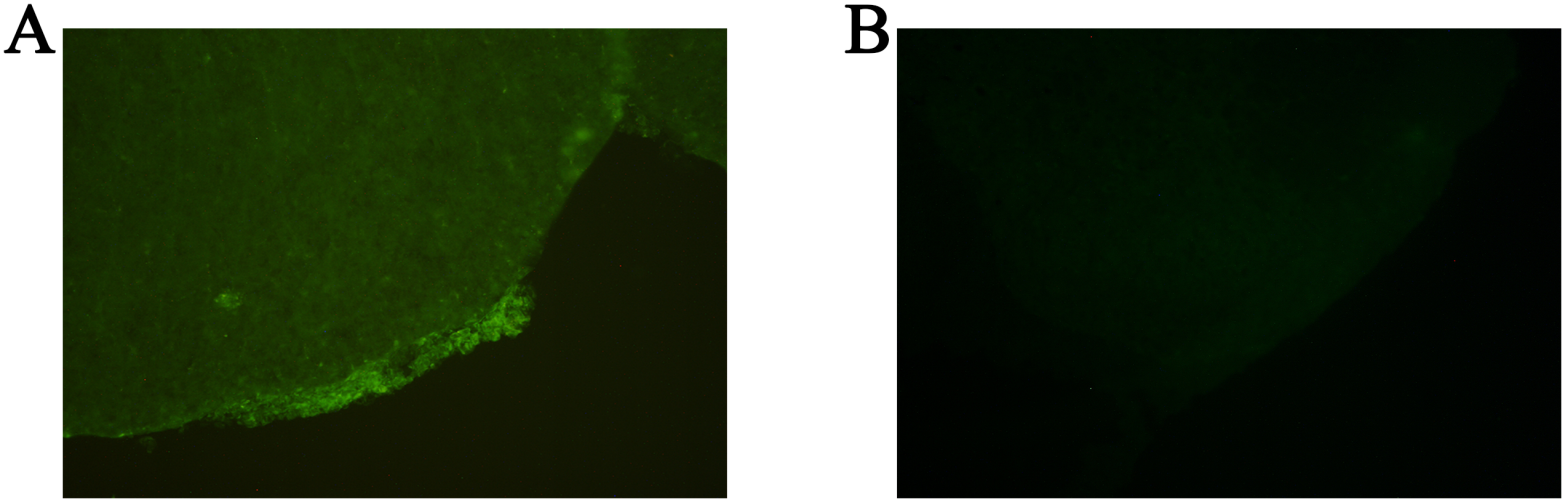
Enhanced green fluorescence protein (EGFP) expression in the meningeal pia mater cells. EGFP expression was located in the meningeal pia mater cells in the Ad-EGFP group after intrathecal injection (see red arrow in the panel A), suggesting that adenovirus was transfected into the same location. This was not observed in the normal saline group as indicated in panel B.

There were no significant changes of the EM2 in CSF on the 1st day as indicated in Fig 2. On the 3rd, 5th, 7th, and 10th day, EM2 in CSF increased significantly in the Ad-EM2 groups compared to the Ad-EGFP group and NS group (^a^P<0.0001). The up-regulation of EM2 in CSF after intrathecal administration of Ad-EM2 indicated an out-secretory expression of EM2. After intrathecal injection, the EGFP expression was detected in the meningeal pia mater cells in the Ad-EGFP group (Fig 3), suggesting that the adenovirus was transfected into the same location after intrathecal injection and the expression of EM2 occurred in an out-secretory manner.

### The alleviation of the morphine withdrawal syndrome

**Fig 4:**
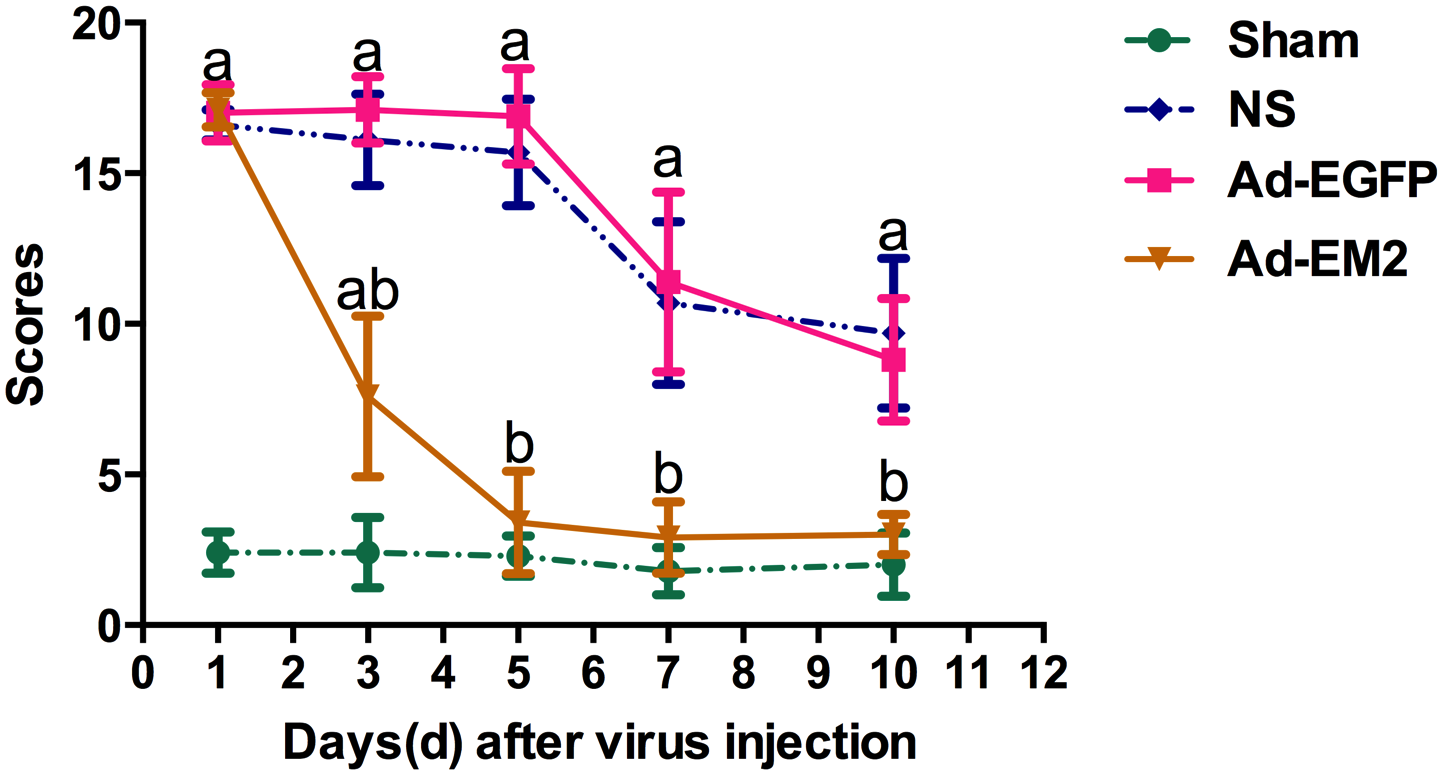
Total score of morphine withdrawal symptoms. On the 3rd, 5th, 7th, and 10th day after intrathecal injection, withdrawal scores were decreased markedly in the Ad-EM2 group compared to that in the Ad-EGFPand NS groups (^b^P<0.0001). Endomorphin-2 was indicated as EM2; adenovirus with EM2 was indicated as Ad-EM2; adenovirus with enhanced green fluorescence protein was indicated as Ad-EGFP; normal saline group with subcutaneous morphine injection and intrathecal NS administration was indicated as NS; Sham group with subcutaneous NS injection and intrathecal NS administration was indicated as Sham.

Compared to the sham group, the scores of the symptoms of morphine withdrawal were significantly higher in the NS and Ad-EGFP group after cessation of morphine (^a^P<0.0001), and the scores were higher in the Ad-EM2 on the 1st, 3rd day (^a^P<0.0001). On the 1st day after intrathecal injection, there were no significant differences for the scores among three groups of dependent rats. On the 3rd, 5th, 7th, and 10th day, withdrawal scores decreased significantly in the Ad-EM2 group compared to the Ad-EGFP and NS groups (^b^P<0.0001) as shown in Fig 4, suggesting that the increase of EM2 in the CSF associated with alleviation of the morphine withdrawal syndrome.

### The change of body weights

**Fig 5:**
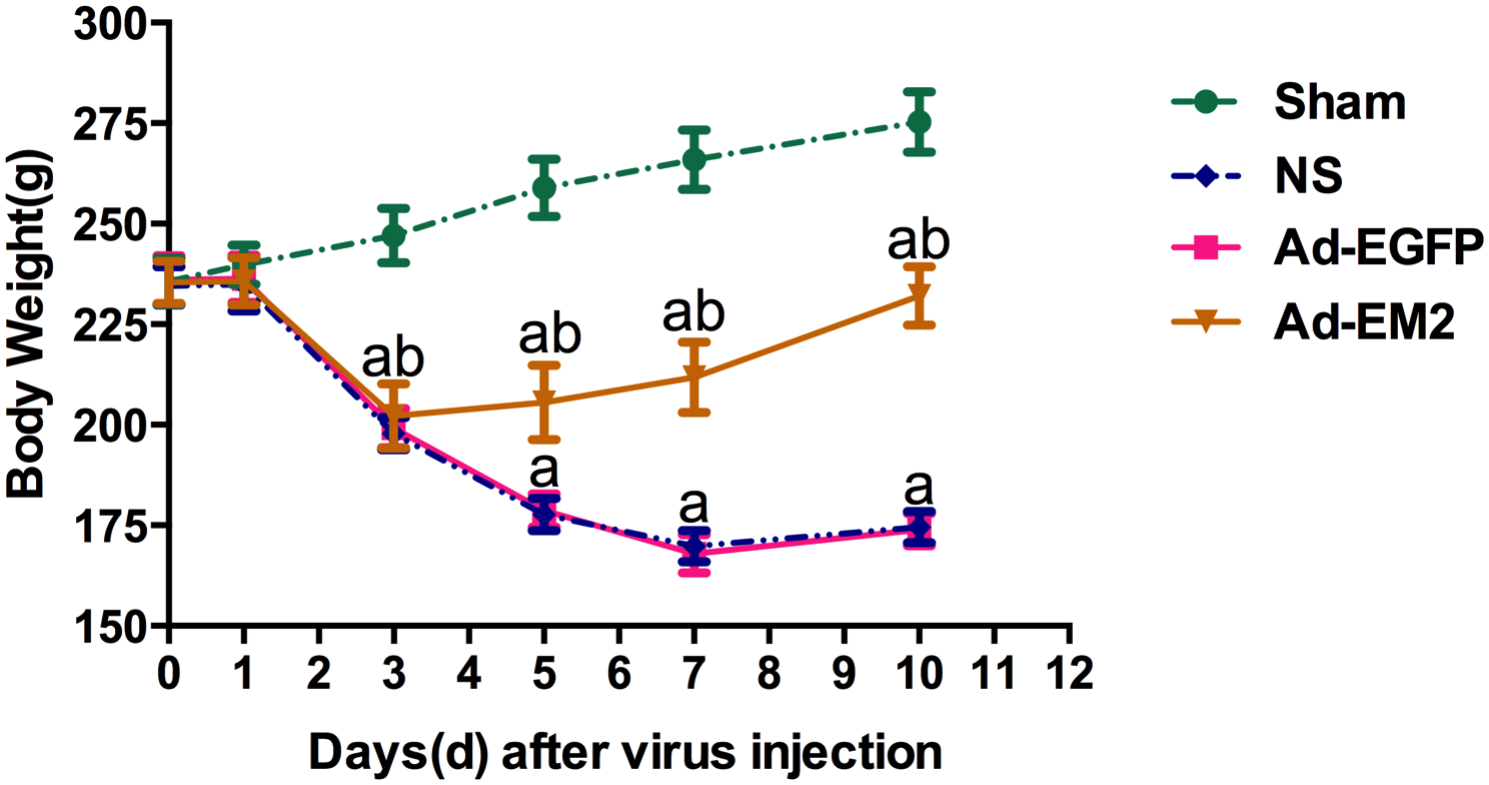
Body weight changes among groups. In the Ad-EM2 group, on the 5th, 7th, and 10th day after intrathecal administration, the body weight increased significantly compared to the Ad-EGFP and the NS groups (^b^P>0.0001), suggesting that EM2 expressed by Ad-EM2 reduced the loss of body weight in morphine dependent rats. Endomorphine-2 was indicated as EM2; adenovirus with EM2 was indicated as Ad-EM2; adenovirus with enhanced green fluorescence protein was indicated as Ad-EGFP; normal saline group with subcutaneous morphine injection and intrathecal NS administration was indicated as NS; Sham group with subcutaneous NS injection and intrathecal NS administration was indicated as Sham.

Loss of body weight was found at 3 days after cessation of morphine in the morphine-dependent rats (Fig 5). Compared to the sham group, there was a significant weight loss in the other three groups receiving morphine injection on the 3rd, 5th, 7th, and 10th day (^a^P<0.0001). In the Ad-EM2 group, the body weight significantly increased compared to the Ad-EGFP and NS groups on the 5th, 7th, and 10th day after intrathecal injection (^b^P<0.0001), suggesting that EM2 expressed by Ad-EM2 associated with the reduction of the loss of body weight in the morphine dependent rats.

## Discussion

In the present study, EM2 gene was introduced into the central nervous system intrathecally via an adenovirus resulting in sustained increase of EM2 in CSF. This led to a significant reduction of the morphine withdrawal syndrome in the morphine dependent rat model.

### The sustained EM2 production

In 1997, EM2 was discovered as an endogenous ligand for MOR [29]. The potent analgesic effect of EM2 has encouraged many studies for potential clinical usage. However, there are two major obstacles for its clinical usage. 1) EM2, with four short amino acid residues, is easily degraded by various enzymes [30] 2) Unknown original gene sequences of EM2 limit the possibility of engineering through transgene methods. In the present study, we bypassed these obstacles by engineering a complex of gene for EM2 and a type I signal peptide of mouse growth factor. A cleavage site of the enzyme, Furin, was introduced for out-secretory expression. Furin cleaves off the signal peptide from the fusion protein and releases EM2 from the membrane of cells [31, 32]. In a neuropathic pain rat model, a single intrathecal injection of the adenovirus carrying Ad-EM2 gene induced 35 days of anti-nociception effect without showing any tolerance or addiction [22]. Thus, this novel Ad-EM2 would be a useful tool for the prolonged release of EM2 for therapeutic purposes.

### Gene therapy of opioid dependence

In opioid dependence, the tone of the endogenous opioid system is too low to restore the deficiency caused by long-term external opioids exposure [33]. After cessation of external opioids, the addict enters an acute withdrawal syndrome due to the shortage of endogenous opioid. Compensation of opioids, such as enkephalins [34], β-endorphin [35] and endomorphin [36], have shown potential applications in treatment of dependence. Using trans-gene technique, dependence related protein such β-endorphin and Cocaine Hydrolase, have been transducted into central nervous system and shown the attenuation of dependence, which results suggest that transduction of substantial mounts of an engineered human protein in or near the cells that are affected in dependence could directly alter the pathophysiologic state [37, 28]. This study demonstrated that EM2 was increased in the CSF 3 days after intrathecal injection of Ad-EM2 and that the acute withdrawal syndrome of morphine was reduced with the increase of EM2. The body mass of the rats also increased after administration of Ad-EM2. These findings supported the hypothesis that EM2 expressed by Ad-EM2 has therapeutic effects for acute opioid withdrawal syndrome.

### The adenoviral vector

The replication deficient adenovirus is a useful vector to deliver genes for neurological disorders [38]. As a tool to deliver EM2, the adenovirus had several advantages. 1) Unlike retroviral vector targeting proliferation cells, the adenoviral vector transfected into both proliferation and non-proliferating cells. It was beneficial for the application of most non-proliferating cells in the nervous system [39]. 2) The protein in CSF delivered by the adenovirus quickly affected neighboring neurons and directly targeted receptors such as mu opioid receptor without neural toxicity. This minimized the dosage and reduced the systemic side effects [40, 41]. 3) The adenovirus did not integrate into the chromosome of the hosts, which could decrease the likelihood of insertion mutation [42, 43]. However, one of the disadvantages of the adenovirus was that it may induce an immune response of the host, which would eliminate the virus gradually [44, 45]. This elimination of the adenovirus resulted in the gradual decrease of EM2 in the CSF [22]. However, this gradual decrease of EM2 could be an advantage of gene therapy, similar to the decreasing dose regimen for methadone [46]. In previous clinical trials, the adenovirus has been used to deliver into the normal brain parenchyma to manage tumors or brain diseases with a safe and successful profile [47, 48]. Thus, such approach could be potentially used to deliver EM2 to manage opioid dependence and withdrawal syndrome.

### The route of administration

For the treatment of acute withdrawal syndrome using adenoviral vectors, the vectors were usually delivered intracerebroventricularly [28] or directly into the brain tissue, in the nucleus accumbens and ventromedial striatum [37]. These methods require complicated procedures and induce additional tissue trauma and potential infections. In the present study, the adenovirus was delivered intrathecally, which provides a safer and more convenient way of administration compared to an intracerebroventricular or brain tissue injection. The adenovirus was detected in the location of the meningeal pia mater cells surrounding the spinal cord, which was consistent in the study of Finegold AA, et al [49]. This alternative route of administration of adenovirus could be a safe and convenient way to manage withdrawal syndrome.

### Limitation of the study

In clinical practice, the levels and timing of EM2 expression are required to be controlled precisely, thus needing an inducible gene expression system such as an RU486 regulating system. Other models such as conditioned place preference model are also required for observation of long-term effect on chronic models. Therefore, further studies are warranted.

## Conclusions

In summary, an engineered EM2 gene delivered to the central nervous system via intrathecal administration reduced withdraw syndrome in an opioid dependent rat model, indicating that this novel engineered EM2 gene could be an alternative therapeutics for management of withdrawal syndrome in opioid dependent subjects.
